# Correction to: ‘Raising the value of research studies in psychological science by increasing the credibility of research reports: The Transparent Psi Project’ (2023) by Kekecs *et al.*

**DOI:** 10.1098/rsos.231080

**Published:** 2023-08-16

**Authors:** Zoltan Kekecs, Bence Palfi, Barnabas Szaszi, Peter Szecsi, Mark Zrubka, Marton Kovacs, Bence E. Bakos, Denis Cousineau, Patrizio Tressoldi, Kathleen Schmidt, Massimo Grassi, Thomas Rhys Evans, Yuki Yamada, Jeremy K. Miller, Huanxu Liu, Fumiya Yonemitsu, Dmitrii Dubrov, Jan Philipp Röer, Marvin Becker, Roxane Schnepper, Atsunori Ariga, Patrícia Arriaga, Raquel Oliveira, Nele Põldver, Kairi Kreegipuu, Braeden Hall, Sera Wiechert, Bruno Verschuere, Kyra Girán, Balazs Aczel

*R. Soc. Open Sci.* **10**, 191375. (Published online 1 February 2023) (doi:10.1098/rsos.191375).

## 1. Summary

This document provides corrections for the paper titled ‘Raising the value of research studies in psychological science by increasing the credibility of research reports: The Transparent Psi Project’ [1]. The corrections are in response to the protocol deviations uncovered by a new research audit conducted on this project by James E. Kennedy [2]. The audit report lists a number of protocol deviations that were missing or were not explicitly listed as such in the original paper. As noted in the new audit report, these protocol deviations are unlikely to have significant influence on the study conclusions, especially given that the study obtained a null result. Nevertheless, if the study would have obtained a positive result (evidence supporting the ESP hypothesis), some of these protocol deviations would have potentially been more impactful casting doubt on such a controversial finding. In this document, corrections are listed for all protocol deviations mentioned in this new audit report.

## 2. Introduction

This document provides corrections for the previously published paper titled ‘Raising the value of research studies in psychological science by increasing the credibility of research reports: The Transparent Psi Project’ [1] available at https://royalsocietypublishing.org/doi/10.1098/rsos.191375. The errors in the paper were uncovered by James E. Kennedy, who undertook a comprehensive audit of the project after its publication. The audit incorporated his experience with research audits in regulated clinical trials. The report resulting from this audit is currently available as a preprint [2] here: https://psyarxiv.com/mytgd/, together with another document detailing the lessons learned and recommendations for the field based on this research audit [3]: https://psyarxiv.com/3wz6f/. The audit report and lessons-learned document cover many methodological topics in addition to the protocol deviations addressed here. The documents may be useful in planning high-quality future research as well as for the full assessment of the Transparent Psi Project.

## 3. Errors, missing information and corrections

The first three cases below were identified in the audit report as potentially significant protocol deviations and the last two cases were described as not significant protocol deviations.

**Error 1**: We note in our paper that ‘Software code running on the server was version controlled and any changes were automatically tracked via git. The IT auditor was able to verify at any time that the software code was unaltered via assessing the tracked changes, and via comparing this to the instance of the code deposited in GitLab at the start of the project’. However, this was not our original intention. Our protocol stated that the data collection software would be automatically synced with GitLab throughout the data collection period, this, keeping the version control more securely at a third-party, instead of relying only on the git record at our server locally. In our paper, this was not explicitly noted as a protocol deviation. Furthermore, in section ‘6.5. Tamper-evident technology for fraud protection’ we note that ‘Specifically, our data were recorded through a custom-made experimental software, which updated its own source code from the git repository's remote master branch every 5 min. This way, the data could not come from any other source than the latest version of the software on the git repository, which keeps an audit trail (version history) recording any changes in the software during data collection’. The preregistered protocol also stated that the research auditors would be able to use the GitLab repository during the study to verify that the data collection software did not have unauthorized alterations. This was our original intended protocol, however, as noted earlier, this was not executed as planned, we only kept a local copy of the git change history instead of pushing it to GitLab in real time, and the research auditors did not have the ability to verify during the study that the software had not been altered. The reason for this protocol deviation was miscommunication with the developer. The original developer handed over the management of the project to a new developer before the project went live, and the intent for the use of GitLab was misunderstood by the new developer.

**Correction 1**: Section ‘6.5. Tamper-evident technology for fraud protection’ incorrectly states that the data collection software updated its own source code from the git repository's remote master branch every 5 min. Instead, contrary to the preregistered protocol, GitLab only recorded the starting state of our software. This should be noted as a protocol deviation. The integrity of the data collection software was verified by a retrospective, highly technical, ad hoc evaluation by the IT auditor, rather than by the more transparent systematic real-time tracking that had been intended.

**Error 2**: One of the missing information noted by the new audit report was related to the failed server access log. Originally, we intended to keep an automated server access log recorded by the server used for data collection, as is standard procedure in a secure computer environment. However, the log configuration was set inappropriately, causing the log file to be overwritten every few days. We reported this issue in our paper, but it is not explicitly noted as a protocol deviation. The reason for the protocol deviation was human error, the developer incorrectly assumed that server logs are kept permanently by default.

**Correction 2**: The failure to keep server access logs for the whole data collection period is in fact a protocol deviation. The retrospective ad hoc evaluation of the data collection server and software by the IT auditor to a significant extent compensated for the lack of the more transparent systematic real-time tracking logs that had been intended.

**Error 3**: An inconsistency in our reporting appeared related to the independence of auditors from the main research team. In ‘2.3.7. External research audit’ section, we stated that ‘An IT auditor and two research auditors independent of the laboratories involved in the study were also involved in the project’. However, later in ‘3.2.4. Audit results, protocol deviations and unexpected events’ section, we note that ‘Potential conflicts of interest may exist: one of the research auditors and the IT auditor work at University of Padova, where we also had a collaborating laboratory. The IT auditor has joint publications with the lead researcher of the University of Padova site’. The reason for this protocol deviation was the lack of available IT auditors. The core research team spent months searching for and negotiating with potential independent IT auditors, but none of the quotes from independent auditors were within the budget of the project. Thus, finally, the independence criteria was relaxed to allow the project to commence.

**Correction 3**: Section ‘2.3.7. External research audit’ incorrectly stated that the IT auditor was independent, since he had ties with one of the collaborating laboratories. This is also a protocol deviation, since we pre-specified recruiting independent auditors.

**Error 4**: According to the laboratory notebooks, there have been a few occasions where the experimental software has been restarted due to time-out because of lost internet connection or due to software crash. This is contrary to the instruction manual to experimenters, which states that the software should not be restarted if the participant is unable to finish a full research session for some reason. This deviation from protocol may have resulted in a very few sessions which were conducted by the same participants. Although it is unclear how much of the data this deviation affects exactly, because it is sometimes unclear from the laboratory notebooks whether the experimental trials have already started when the crash happened, it is likely very small compared with the total number of research sessions (2115 analysed sessions), the auditor identified at least five sessions: rows 196, 207, 208, 220, 290 in the laboratory notebook spreadsheet. Even though this may have caused a small deviation from the intended protocol, as stated in the new audit report, these cases cannot create a false-positive ESP effect. The reason for this protocol deviation is likely human error, that is, some experimenters did not remember the instruction that software should not be restarted if the participant is unable to finish a full research session.

**Correction 4**: A small number (at least five out of 2115) of experimental sessions were restarted, thus, potentially leading to data from the same participant occurring in two research sessions instead of one. This is a protocol deviation. However, due to the small number of occurrences and the nature of the data analysis involved, this has a negligible effect on the results, and no effect on the conclusions of the study.

**Error 5**: Section ‘2.8. Inclusion of data in analysis’ notes that data from test sessions were excluded from data collection and that ‘The experimenter ID(s) of the test account(s) were specified in the preregistered analysis code’. However, it is not noted that two IDs to exclude were added 10 day after the data collection was started. These IDs were test account IDs that were missing from the originally preregistered code. When we noticed this shortly after data collection started, we added these missing test account IDs to the registered code. This change to the code was documented on GitHub. The new audit report notes that ‘The excluded data for the two added laboratory IDs were only 81 records widely dispersed throughout the study and could not affect the study conclusions. The records appear consistent with test data, although it is not clear why such testing was done or who did it’.

**Correction 5**: Section ‘2.8. Inclusion of data in analysis’ incorrectly notes that ‘The experimenter ID(s) of the test account(s) were specified in the preregistered analysis code’. Two missing IDs were added shortly after the start of data collection, this being clearly documented in the repository of the analysis code: https://github.com/kekecsz/Transparent_psi_RR_materials/commit/4e4e2e4df561205fc707837add79c9e5bcbc4eb3. This affects only 81 rows in the raw dataset, 19 of these rows include trials from the ‘erotic’ condition, by comparison, the results are based on analysing 37 836 trials from the ‘erotic’ condition. Since these were test trials, the results and conclusions are not affected by this issue. However, the fact that this change was made was missing from the paper and should have been noted.
